# Recipient’s Genetic R702W *NOD2* Variant Is Associated with an Increased Risk of Bacterial Infections after Orthotopic Liver Transplantation

**DOI:** 10.1371/journal.pone.0072617

**Published:** 2013-08-19

**Authors:** Marcel Janse, Bert-Jan F. de Rooij, Bart van Hoek, Arie P. van den Berg, Robert J. Porte, Hans Blokzijl, Minneke J. Coenraad, Bouke G. Hepkema, Alexander F. Schaapherder, Jan Ringers, Rinse K. Weersma, Hein W. Verspaget

**Affiliations:** 1 Department of Gastroenterology and Hepatology, University of Groningen and University Medical Center Groningen, Groningen, The Netherlands; 2 Department of Gastroenterology and Hepatology, Leiden University Medical Center, Leiden, The Netherlands; 3 Department of Hepatobiliary Surgery and Liver Transplantation, University of Groningen and University Medical Center Groningen, Groningen, The Netherlands; 4 Department of Laboratory Medicine, University of Groningen and University Medical Center Groningen, Groningen, The Netherlands; 5 Department of Surgery, Leiden University Medical Center, Leiden, The Netherlands; Centro di Riferimento Oncologico, IRCCS National Cancer Institute, Italy

## Abstract

**Introduction:**

Orthotopic liver transplantation (OLT) is accompanied by a significant postoperative infection risk. Immunosuppression to prevent rejection increases the susceptibility to infections, mainly by impairing the adaptive immune system. Genetic polymorphisms in the lectin complement pathway of the donor have recently been identified as important risk determinants of clinically significant bacterial infection (CSI) after OLT. Another genetic factor involved in innate immunity is *NOD2*, which was reported to be associated with increased risk of spontaneous bacterial peritonitis in cirrhotic patients.

**Methods:**

We assessed association of three genetic *NOD2* variants (R702W, G908R and 3020insC) with increased risk of CSI after OLT. 288 OLT recipient-donor pairs from two tertiary referral centers were genotyped for the three *NOD2* variants. The probability of CSI in relation to *NOD2* gene variants was determined with cumulative incidence curves and log-rank analysis.

**Results:**

The R702W *NOD2* variant in the recipient was associated with CSI after OLT. Eight out of 15 (53.3%) individuals with a mutated genotype compared to 80/273 (29.3%) with wild type genotype developed CSI (p=0.027, univariate cox regression), illustrated by a higher frequency of CSI after OLT over time (p=0.0003, log rank analysis). Multivariate analysis (including the donor lectin complement pathway profile) showed independence of this R702W *NOD2* association from other risk factors (HR 2.0; p=0.04). The other *NOD2* variants, G908R and 3020insC, in the recipient were not associated with CSI. There was no association with CSI after OLT for any of the *NOD2* variants in the donor.

**Conclusion:**

The mutated *NOD2* R702W genotype in the recipient is independently associated with an increased risk of bacterial infections after liver transplantation, indicating a predisposing role for this genetic factor impairing the recipient’s innate immune system.

## Introduction

Results of liver transplantations have steadily improved over the last decades with 5-years patient survival at 70%-80% in recent years. This success is attributable to improved operative techniques, earlier detection and treatment of post operative complications and new immunosuppressive drugs [1]. However, infections still represent an important clinical problem after orthotopic liver transplantation (OLT) with significant patient morbidity and mortality [2]. There are several contributing factors for this high infection risk, for example the immunosuppressive agents that prevent rejection increase the susceptibility to infections, mainly by interference with the acquired immune system. In addition, previous studies showed that genetic polymorphisms in the lectin complement pathway of the donor also contributes significantly to the risk of infection after liver transplantation [3,4]. With the current study we assessed whether genetic polymorphisms in *NOD2* (nucleotide-binding oligomerization domain containing 2), also pivotal in the innate immunity, are associated with infectious complications and mortality after OLT.

*NOD2* is an intracellular receptor, recognizing bacterial peptidoglycan, present in macrophages, dendritic cells and certain intestinal epithelial cells, including paneth cells, that play an important role in innate immunity [5–7]. Three genetic *NOD2* variants are known to be strongly associated with Crohn’s disease [8,9]. The same *NOD2* variants are also involved in several infectious conditions: an increase in sepsis related mortality in the ICU has been described [10] and recently the occurrence of spontaneous bacterial peritonitis in liver cirrhosis patients was reported to be increased among *NOD2*-carriers and associated with reduced survival [11]. *NOD2* was also found to be associated with increased risk of graft versus host disease and transplant related mortality in haematopoietic stem cell transplantation [12], and in kidney transplantation mutated *NOD2* haplotypes are associated with higher rates of death with functioning graft [13].

Based on the above we hypothesized that genetic variants in *NOD2* could be associated with increased risk of bacterial infections after liver transplantation, potentially leading to impaired graft- and patient survival.

## Methods

The study was approved by the Medical Ethics Committee from the University Medical Center Groningen, the Netherlands and the Medical Ethics Committee from the Leiden University Medical Center, the Netherlands. From all patients written informed consent was obtained, all in compliance with the Helsinki Declaration

### Study population

The study population consisted of 307 OLT recipient-donor pairs. From both participating centers only those patients whose DNA was available from both donor and recipient, and who had at least 7 days of follow-up after liver transplantation were included. 140 patients received OLT at the Leiden University Medical Center (LUMC) in the Netherlands between 1992 and 2005, and 167 patients received OLT at the University Medical Center Groningen (UMCG) in the Netherlands between 2000 and 2005. The cohort has recently been described in a previous study [4].

The LUMC patients received standard immunosuppressive therapy consisting of corticosteroids, a calcineurin inhibitor (i.e., cyclosporine or tacrolimus) with or without mycophenolate mofetil or azathioprine and/or basiliximab. The UMCG patients received standard immunosuppressive therapy consisting of basiliximab combined with a calcineurin inhibitor with or without corticosteriods and/or mycophenolate mofetil. Until 2001 azathioprine was used instead of mycophenolate mofetil in case of impaired renal function, and basiliximab on day 0 and 4 was introduced in 2001. All patients received 24 hours of prophylactic antibiotics intravenously: gentamycin, cefuroxim, penicillin G, and metronidazol in the LUMC, amoxicillin-clavulanate and ciprofloxacin in the UMCG. In addition, the LUMC patients received 3 weeks of selective digestive tract decontamination (polymyxin/neomycin, norfloxacin, and amfotericin B) after OLT. After surgery, all patients were intensively monitored according to standardized protocols for any infection, rejection, or poor function of the new liver. After hospital discharge, frequent regular visits and standard procedures in case of suspicion of an infection after OLT, including additional visits upon febrile temperature, are operational in both transplant centers.

### Clinical Variables

The general patient database, original patient reports, transplantation databases, and microbiology records were evaluated to identify episodes of clinical and laboratory-confirmed bacterial infections within the first year after transplantation. The identified infections were considered clinically significant bacterial infections (CSI) when they complied with the Centers for Disease Control and Prevention criteria for diagnosing infection [14]. All infections found were categorized into sepsis (including symptomatic urinary tract infection - urosepsis), pneumonia; and intra-abdominal infections, i.e., cholangitis and peritonitis.

Demographic and clinicopathological characteristics of the recipient at the time of OLT (age, sex, indication for liver transplantation, Child-Pugh classification, and laboratory Model for End-Stage Liver Disease [MELD] score), donor information (age, sex), and posttransplant follow-up data (immunosuppressive regimen, acute cellular rejection according to the Banff scheme [15]) were also collected from the transplantation databases. Clinical characteristics of donors and recipients are shown in Table 1.

**Table 1:** Characteristics of all Eligible Orthotopic Liver Transplant Recipients and Donors in the Study (N=307).

**Variable**	
Age (median, IQR)	
Recipient	51 (41-56)
Donor	46 (34-55)
Gender donor/recipient, N (%)	
Male/male	105 (34.2)
Male/female	52 (16.9)
Female/male	85 (27.7)
Female/female	65 (21.2)
Primary liver disease, N (%)	
Viral	59 (19.2)
Alcoholic	46 (15.0)
Cholestatic	79 (25.7)
Other disease^§^	123 (40.1)
Child-Pugh class, N (%)	
A	67 (21.8)
B	138 (45.0)
C	102 (33.2)
Lab MELD score (median, IQR)	15 (11-21)
Donor-type, N (%)	
Donation after cardiac death	24 (7.8)
Donation after brain death	283 (92.2)
Maintenance immunosuppression, N (%)	
Prednisone / CNI / basiliximab (+ MMF)	206 (67.1)
Prednisone / CNI (+ azathioprine)	101 (32.9)
Acute cellular rejection, N (%)	
Yes	107 (34.9)
No	200 (65.1)
Bacterial infection, N (%)	94 (30.6)
LCBI	52 (55.3)
PNEU	10 (10.6)
IAB	32 (34.0)

IQR denotes interquartile range; MELD, Model for End-Stage Liver Disease; CNI, calcineurin inhibitors; MMF, mycophenolate mofetil; LCBI, laboratory-confirmed bloodstream infection; PNEU, pneumonia; IAB, intra-abdominal infection.

^§^ Other diseases included predominately autoimmune hepatitis, cryptogenic cirrhosis and metabolic disorders.

### Genotyping of the NOD2 variants

Genomic DNA was extracted routinely from peripheral blood and/or tissue samples.

All donors and recipients were genotyped for the *NOD2* SNPS rs2066844 (R702W), rs2066845 (G908R), and rs2066847 (frame-shift mutation 1007fs, or 3020insC) by means of *Applied Biosystems Taqman* (ABI 7900 HT)^*®*^ technology according to the manufacturer’s recommendations at the Laboratory of Genetics at the UMCG in January 2012. For rs2066844 and rs2066845 pre-made assays were provided by applied biosystems (AB ID:C_11711717468_20 and AB ID C_11717466_20 respectively). The genotyping assay for rs2066847 was custom developed by Applied Biosystems (forward primer GTCCAATAACTGCATCACCTACCT; reverse primer CAGACTTCCAGGATGGTGTCATTC; VIC-labeled MGB probe CAGGCCCCTTGAAAG; FAM-labeled MGB probe CAGGCCCTTGAAAG).

The results of the genotyping assay were analyzed using the SDS 2.3 software compatible with the *Applied Biosystems Taqman^®^*. Results obtained from SDS were exported to excel and SPSS for further statistical analyses.

### Statistical analysis

Associations between baseline characteristics of the liver transplant recipients, donors, and posttransplant follow-up data and CSI were performed with the use of univariate Cox regression models. The probability of clinically significant infection within the first year after transplantation in relation to *NOD2* gene variants was determined with cumulative incidence curves using Kaplan-Meier analysis, and the differences between groups were assessed by log-rank test. Patients were censored at the date of the last follow-up, death, or liver retransplantation. The multivariate Cox proportional hazards regression analysis was used to evaluate the independence of the potential association of R702W, G908R and 3020insC with infection. Factors associated with infection with a P value of less than 0.15 in the univariate analysis were entered in the multivariate model and non-significant factors were removed by means of a backward-selection procedure. In this multivariate analysis it was also assessed whether associations were independent from previously described associated genetic lectin pathway variants in the donor [4]. Results were considered statistically significant when P values were <0.05. Bonferroni correction for the number of SNPs tested was not performed because SNPs were selected on the basis of a deducible hypothesis. All analyses were performed with the SPSS statistical software package (version 16.02; SPSS, Inc., Chicago, IL).

## Results

### Prevalence of NOD2 variants in recipients and donors

Overall more than 95% of all individuals were successfully genotyped for the three *NOD2* variants R702W (rs2066844), G908R (rs2066845) and 3020insC (2066847). In individuals with all variants successfully genotyped any *NOD2* variant was present in 39/287 (13.6%) of the recipients of an OLT and in 54/289 (18.7%) of the liver donors (p=0.15).

The minor allele frequency (MAF) in R702W was higher in donors compared to recipients (5.1% and 2.8%, p=0.050). In G908R MAF was 2.9% in recipients and 2.0% in donors (p=0.35) and in 3020insC MAF was 1.2% in recipients and 2.2% in donors (p=0.18). Table S1 illustrates genotype distribution for all three SNPs in recipients and donors stratified for occurrence of CSI.

### NOD2 variants in recipients and donors in relation to infectious complications

**Figure 1:**
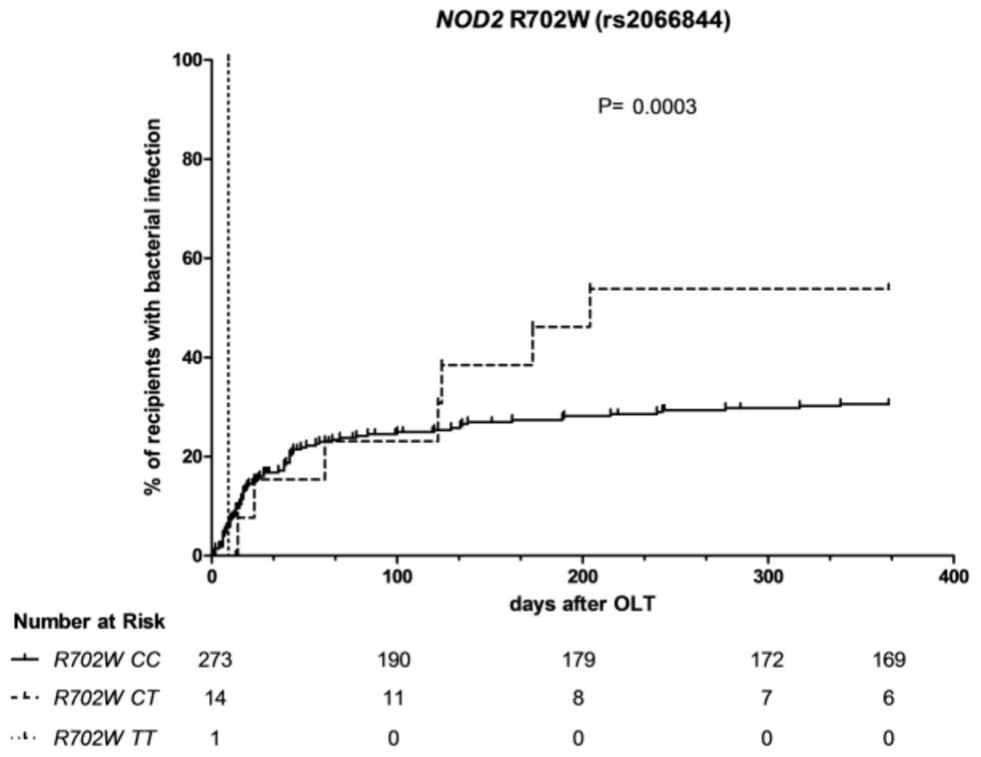
Development of clinically significant infection over time in relation to the recipient *NOD2* R702W genotype. Occurrence of clinically significant infection in days after OLT in relation to the recipient *NOD2* R702W genotype. CC = wildtype, TT homozygote for mutation, CT = heterozygote. The numbers below the Kaplan – Meier curves represent the number of individuals ‘at risk’ per genotype at particular time points after transplantation.

R702W in the recipients, successfully determined in 288 cases, was significantly associated with the development of infectious complications after liver transplantation. In recipients with one or two risk alleles (genotype CT and genotype TT) for R702W the CSI frequency was 53.3% (8 out of 15) as opposed to 29.3% (80 out of 273) in recipients with the wildtype (CC) genotype (HR 2.1, p = 0.03, univariate cox regression). The development of a CSI over time was also significantly higher in individuals with one or to mutated alleles in R702W (p < 0.001, log rank analysis, Figure 1.). One recipient carrying the TT genotype developed infection early after transplantation. When grouping the two risk genotypes (CT and TT) together, the association was borderline nonsignificant (p=0.070, univariate cox regression; p = 0.063, log rank analysis; p=0.096 in multivariate analysis). There was no significant relation between site-specific infections (i.e., laboratory-confirmed bloodstream infection, pneumonia or intra-abdominal infection) and mutated alleles in R702W (data not shown).

The association of recipient *NOD2* R702W showed the same trend direction in both independent sub cohorts. In cohort 1 the only individual with TT genotype and two out of six (33.3%) individuals with CT genotype compared to 33 out of 151 (21.9%) individuals with CC genotype developed infection after transplantation (p < 0.001, log rank analysis). In cohort 2 the similar, although not statistically significant, trend was observed, i.e., five out of eight (62.5%) individuals with CT genotype developed infection after OLT compared to 47 out of 122 (38.5%) individuals with the CC genotype (p = 0.40, log rank analysis).

In the other two *NOD2* variants, G908R and 3020insC, recipient genotypes were equally distributed between patients that did or did not develop a CSI (p = 0.13 and p = 0.96 respectively). Regarding the *donor* genotypes there was no significant association with the number of CSI after transplantation for any of the three *NOD2* variants (R702W p = 0.26, G908R p = 0.16, 3020insC p = 0.65; Table S1).

We also assessed whether there was a difference in occurrence of CSI in individuals carrying one or more of either *NOD2* variants in the recipient or donor. Twelve out of 39 (30.8%) individuals with one or more *NOD2* variant in the recipient developed CSI compared to 76 out of 248 (30.6%) individuals without recipient *NOD2* variant (p = 0.97). Similarly, regarding the donor *NOD2* status no difference was observed in the occurrence of CSI: 14 out of 54 (25.9%) individuals with one or more *NOD2* variant in the donor developed CSI compared to 70 out of 235 (29.8%) individuals without donor *NOD2* variant (p = 0.48)

### Multivariate analysis

Based on the observed *NOD2* association a multivariate analysis was performed to assess whether the R702W risk allele was a risk factor for the development of CSI independent from recently described associations in the lectin pathway donor profile and other potential risk factors. This analysis showed that the association of the minor allele of *NOD2* R702W in the recipient is indeed independent from the lectin pathway profile of the donor and the only other significant contributing factor, namely the gender of the recipient donor pair (HR 2.0, p = 0.04; Table 2)

**Table 2:** Univariate and Multivariate Analysis to Infection risk after orthotopic liver transplantation (N=288).

			Univariate			Multivariate	
**Variable**	Bacterial infection % (N)	HR	CI (95%)	P Value	HR	CI (95%)	P Value
Age							
Recipient	30.6 (88/288)	0.99	0.98-1.0	0.37			
Donor	30.6 (88/288)	0.99	0.98-1.0	0.11			
Gender recipiënt				**0.02**			**0.02**
Male/male	40.8 (40/98)	2.6	1.4-5.0	0.004	2.6	1.4-5.0	0.004
Male/female	30.0 (15/50)	1.8	0.84-3.8	0.13	1.5	0.68-3.2	0.32
Female/male	27.6 (21/76)	1.6	0.79-3.3	0.19	1.5	0.71-3.0	0.30
Female/female	18.8 (12/64)	ref			ref		
Primary liver disease				0.49			
Viral	37.5 (21/56)	1.5	0.86-2.6				
Alcoholic	26.3 (10/38)	1.1	0.52-2.1				
Cholestatic	33.3 (25/75)	1.3	0.77-2.2				
Other disease^§^	26.9 (32/119)	ref					
Child-Pugh class				0.14			
C	36.8 (35/95)	1.8	0.99-3.4	0.05			
B	30.0 (39/116)	1.4	0.76-2.6	0.29			
A	22.2 (14/63)	ref					
Lab MELD score	31 (88/288)	1.0	0.97-1.0	0.72			
Donor-type							
Donation after cardiac death	37.5 (9/24)	1.3	0.65-2.6	0.47			
Donation after brain death	29.9 (79/264)	ref					
Immunosuppression							
Prednisone/CNI/basiliximab (+ MMF)	28.1 (55/196)	0.74	0.48-1.1	0.18			
Prednisone/CNI (+ azathioprine)	35.9 (33/92)	ref					
Acute cellular rejection							
Yes	24.0 (24/100)	0.60	0.38-0.95	0.03			
No	34.0 (64/188)	ref					
CMV seropositivity				0.74			
D+/R+	26.4 (23/87)	0.79	0.42-1.50	0.47			
D+/R-	30.8 (12/39)	0.95	0.45-2.01	0.89			
D-/R+	32.5 (37/114)	1.1	0.59-1.90	0.85			
D-/R-	33.3 (16/48)	ref					
Lectin complement pathway donor profile^‡^		1.8	1.4-2.3	**<0.001**	1.8	1.4-2.3	**<0.001**
*3 variants*	63.2 (12/19)	*4.1*	*1.6-10.4*	*0.003*	*4.1*	*1.6-10.5*	*0.003*
*2 variants*	39.8 (41/103)	*2.5*	*1.1-5.5*	*0.03*	*2.3*	*1.0-5.1*	*0.047*
*1 variant*	21.7 (28/129)	*1.2*	*0.5-2.6*	*0.73*	*1.1*	*0.47-2.5*	*0.86*
*0 variants*	18.9 (7/37)	*ref*			*ref*		
Recipient R702W		2.1	1.1-4.2	**0.03**	2.0	1.0-4.0	**0.040**
*TT*	100 (1/1)	*18.1*	*2.4-136.6*	*0.005*			
*CT*	50.0 (7/14)	*1.7*	*0.80-3.8*	*0.16*			
*CC*	29.3 (80/273)	*ref*					

HR denotes hazard ratio; CI, confidence interval; MELD, Model for End-Stage Liver Disease; CNI, calcineurin inhibitors; MMF, mycophenolate mofetil

^‡^The presence of donor-recipient genotypic groups associated with bacterial infections within the individual were clustered as lectin pathway donor profile; MBL2: XA/O and O/O donor and A/A and YA/O recipient; FCN2: CT or TT donor and or recipient; MASP2: AA donor and or recipient

### NOD2 variants in recipient and donor in relation to patient survival

None of the *NOD2* variants in donor or recipient was found to be associated with patient survival the first year after OLT. In total 27 patients deceased within one year after transplantation: one recipient carried the R702W variant, two recipients carried the G908R variant and two recipients carried the 3020insC variant. R702W, G908R and 3020insC was present in five, zero and one donor(s) respectively.

## Discussion

In the current study it was found that the existence of the *NOD2* variant R702W in the recipient is associated with an increased risk of bacterial infections after liver transplantation. This association was found to be independent of clinical variables, like gender, that are associated with increased bacterial infections. Furthermore, this *NOD2* R702W association was shown to be independent of the previously described infection risk mutations in the lectin complement pathway of the donor liver [4].

Liver transplant recipients with one or two risk alleles for R702W (genotype CT and genotype TT) in the *recipient* developed more often a clinically significant infection, i.e., sepsis, pneumonia or abdominal infection, than individuals with a wild type genotype. It is interesting to note that one individual with homozygote TT risk for *NOD2* R702W variant in the recipient developed infection early after transplantation. The recipients with heterozygote CT risk genotype developed infection more than three months after transplantation. We observed no association with *NOD2* variants in recipient or donor with patient survival in the first year after liver transplantation.

The risk of infections after transplantation of a solid organ is the resultant of the net state of an impaired immune defence. Multiple factors contribute to the increased risk of infections, including the outcome of transplantation with or without technical complications, graft dysfunction and the intensity of immunosuppression [16,17]. Genetic predisposition of recipient and/or donor organ influencing the innate immune system is likely to play a central role in further increasing the risk of infection. For example, we recently reported that genetic variants in the lectin complement pathway of the donor increases the risk of CSI after transplantation. Components of the lectin pathway of complement activation, including mannan binding lectin 2 (MBL2), ficolin 2 (FCN2) and Mannan-binding lectin serine protease 2 (MASP2), are primarily produced in the liver. Therefore, functional SNPs in these polymorphic genes present in the donor liver would lead to reduced complement activation and opsonisation resulting in an increased infection risk. We now show that the *NOD2* R702W variant in the recipient is another innate risk factor for bacterial infections after OLT. *NOD2* is a critical component of the intestinal immunological barrier. In the initial period after transplantation this intestinal barrier is already compromised due to the postoperative state of the patient [18,19]. Furthermore, bacterial translocation in cirrhosis is increased due to structural changes of the intestinal mucosa, including dilatation of intercellular space and vascular congestion as well as mucosal oxidative damage [20], and probably these structural changes also contribute to a decreased barrier function during the recovery phase of the gut post transplantation.

As an intracellular receptor *NOD2* (nucleotide-binding oligomerization domain containing 2) recognizes bacterial peptidoglycans. This receptor is present in macrophages, dendritic cells and certain intestinal epithelial cells, including Paneth cells, and is known to play an important role in the innate immunity. The three *NOD2* variants R702W, G908R, 3020insC are all located in the coding region of *NOD2* and lead to an altered amino acid sequence, either due to non-synonymous SNPs that results in amino acid exchanges or through an insertion that results in a frame-shift mutation. Mutations in *NOD2* hence lead to a decreased activation of the NK-κB pathway upon stimulation by bacterial peptidoglycans [8,9]. *NOD2* is also involved in modulation of Toll-like receptor (TLR) signaling [21], and regarding this latter the recipient *TLR2* R753Q variant has also been associated with presentation with septic shock and infection recurrence with gram-positive bacterial infections after OLT [22]. The observation that this *NOD2* R702W variant is associated with CSI after OLT independently from the lectin-binding complement activation system also indicates that there might be an interplay between these antibacterial pathways which merits further functional studies.

Our findings complement the earlier identification of *NOD2* being a risk factor in multiple infectious conditions and transplantation related complications. *NOD2* has, for instance, been reported to be associated with increased sepsis-related mortality in patients admitted to an Intensive Care Unit [10]. Another recent study reported an increased risk of spontaneous bacterial peritonitis (SBP) in cirrhotic patients carrying *NOD2* variants accompanied by a reduced patient survival time after SBP [11]. The same group also reported an increased susceptibility towards SBP in patients carrying *TLR2* polymorphisms [23]. In transplant literature *NOD2* has been linked to increased risk of graft versus host disease and transplant-related mortality in hematopoietic stem cell transplantation [12,24]. However, other reports could not confirm these results [25]. In kidney transplantation mutated *NOD2* haplotypes are associated with higher rates of death with functioning graft, but not with rejection and graft failure [13]. Fishbein et al. found that Crohn’s disease-associated *NOD2* variants were associated with decreased graft and patient survival after small bowel transplantation [26]. Other studies on small bowel transplantation were inconsistent [27], although one study confirmed the R702W variant, the same variant that was related to CSI in the current study, was associated with early rejection and decreased patient survival [28].

There is an increasing interest in the use of biomarkers as predictors for outcome in solid organ transplantation. Various studies tried to estimate or predict the occurrence of rejection and chronic allograft damage by correlating genomic, transcriptomic and proteomic information from donor and recipient with clinical phenotypes. Genomic analysis, for instance, revealed that mutations in the innate immune system protein Toll-like receptors in donor and/or recipient were associated with reduced risk and severity of allograft rejection in liver, lung and kidney [29]. With increasing knowledge about factors involved in the risk of developing infections after liver transplantation such biomarkers might also be a useful tool to identify patients at risk for infections after transplantation. Especially considering the relatively high prevalence of bacterial infections after liver transplantation and the considerable impact on patient morbidity and mortality such predictors could have a considerable clinical impact. The current study adds to that knowledge by reporting the association of the *NOD2* R702W variant in the recipient with increased risk of bacterial infections after liver transplantation. However, the frequencies of the three *NOD2* variants we found are low but comparable to the frequencies reported in the literature [30] and implicates difficult assessment of association of the variants with clinical outcomes.

In conclusion, the mutated *NOD2* R702W genotype in the recipient is associated with an increased risk of bacterial infections after liver transplantation. This association is independent of clinical variables and mutations in the donor liver of the lectin complement pathway. This indicates a possible predisposing role for this recipient *NOD2* genetic risk factor impairing the innate immune system after orthotopic liver transplantation.

## Supporting Information

Table S1 **Frequencies of NOD2 Polymorphisms in Orthotopic Liver Transplant Recipients and Donors.** HR denotes hazard ratio.
* Recipient: undetermined 19/307 cases (6.2%); donor: undetermined 12/307 cases (3.2%); overall 29/614= 4.7%.
** Recipient: undetermined 15/307 cases (4.9%); donor: undetermined 11/307 cases (3.6%); overall 26/614= 4.2%.
*** Recipient: undetermined 10/307 cases (3.3%); donor: undetermined 11/307 cases (3.6%); overall 21/614= 3.4%.
(DOC)
