# Supplementary material for: Recipient’s Genetic R702W NOD2 Variant Is Associated with an Increased Risk of Bacterial Infections after Orthotopic Liver Transplantation
Source: PLoS One. 2013 Aug 19;8(8):e72617. doi: 10.1371/journal.pone.0072617 (PMC3747080; doi:10.1371/journal.pone.0072617)
Supplement: Table S1 — Frequencies of NOD2 Polymorphisms in Orthotopic Liver Transplant Recipients and Donors. HR denotes hazard ratio. * Recipient: undetermined 19/307 cases (6.2%); donor: undetermined 12/307 cases (3.2%); overall 29/614= 4.7%. ** Recipient: undetermined 15/307 cases (4.9%); donor: undetermined 11/307 cases (3.6%); overall 26/614= 4.2%. *** Recipient: undetermined 10/307 cases (3.3%); donor: undetermined 11/307 cases (3.6%); overall 21/614= 3.4%. (DOC) [file pone.0072617.s001.doc]

**Table S1.** Frequencies of *NOD2* Polymorphisms in Orthotopic Liver Transplant Recipients and Donors

|  |  |  |  |  | |  |  |  | | |  |
| --- | --- | --- | --- | --- | --- | --- | --- | --- | --- | --- | --- |
|  |  |  | **Recipient** | |  | | **Donor** | |  | | |
| **dbSNP ID** | **SNP** | **Genotype** | **CSI** | **No CSI** | **HR** | **P value** | **CSI** | **No CSI** | **HR** | **P value** | |
| ***NOD2*** |  |  |  |  |  |  |  |  |  |  | |
| R702W* | R702W | C🡪T |  |  | 2.128 | **0.0270** |  |  | 1.401 | 0.264 | |
|  |  | CC | 80 | 193 |  |  | 75 | 191 |  |  | |
|  |  | CT | 7 | 7 |  |  | 11 | 17 |  |  | |
|  |  | TT | 1 | 0 |  |  | 0 | 1 |  |  | |
| G908R** | R908R | G🡪C |  |  | 0.338 | 0.130 |  |  | 0.046 | 0.162 | |
|  |  | GG | 84 | 191 |  |  | 87 | 197 |  |  | |
|  |  | CG | 2 | 15 |  |  | 0 | 12 |  |  | |
|  |  | CC | 0 | 0 |  |  | 0 | 0 |  |  | |
| 3020insC*** | c.3020insC |  |  |  | 0.962 | 0.957 |  |  | 0.763 | 0.645 | |
|  |  | neg | 89 | 201 |  |  | 84 | 199 |  |  | |
|  |  | pos | 2 | 5 |  |  | 3 | 10 |  |  | |
